# Extracts of the Algerian Fungus *Phlegmacium herculeum*: Chemical Analysis, Antioxidant, Antibacterial, and Cytotoxicity Evaluation

**DOI:** 10.3390/jof11120894

**Published:** 2025-12-18

**Authors:** Roukia Zatout, Stefania Garzoli, Lounis Youcef Khodja, Ouided Abdelaziz, Maria Michela Salvatore, Anna Andolfi, Marco Masi, Alessio Cimmino

**Affiliations:** 1 Department of Microbial Biotechnology, Faculty of Natural and Life Science, University of Blida 1, Ouled Yaich, Blida 09000, Algeria; 2 Department of Chemistry and Technologies of Drug, Sapienza University, 00185 Rome, Italy; 3 Laboratoire de Recherche en Écologie et en Environnement, Faculté des Sciences de la Nature et de la Vie, Université de Béjaia, Béjaia 06000, Algeria; 4 Department of Plant Biology and Ecology, University of Sétif 1, Setif 19000, Algeria; 5 Laboratoire de Biochimie Appliqué, Département de Biochimie et Biologie Cellulaire et Moléculaire, Faculté des Sciences de la Nature et de la Vie, Université Frères Mentouri, Constantine 1, Route d’Ain el Bey, Constantine 25017, Algeria; 6 Department of Veterinary Medicine and Animal Production, University of Naples Federico II, 80137 Naples, Italy; 7 Department of Chemical Sciences, University of Naples Federico II, 80126 Naples, Italy

**Keywords:** *Phlegmacium herculeum*, molecular identification, phenolic compounds, volatile compounds, antioxidant activity, antibacterial activity, cytotoxicity, ectomycorrhizal fungi

## Abstract

This study reports the first molecularly confirmed occurrence of *Phlegmacium herculeum* in Algeria, identified through morphological features and ITS sequence analysis (GenBank accession: PQ133121). Phytochemical profiling revealed a diverse composition of metabolites. SPME–GC–MS analysis detected volatile aldehydes (butanal, pentanal), organic acids (butanoic, pentanoic), terpenoids (limonene, 1,8-cineole), phenolics, and long-chain alkanes. Furthermore, the macrofungus has been extracted with organic solvents, and the obtained extracts have been analyzed via NMR and GC–MS, revealing the presence of organic acids (lactic, succinic, azelaic), fatty acids (palmitic, linoleic), and phenolic acids (protocatechuic, 4-hydroxybenzoic). DPPH-based analysis indicated that the antioxidant response of the crude extracts strengthened as the dose increased, with the ethyl acetate (EtOAc) extract exhibiting the highest inhibition and lowest IC_50_, attributed to its rich phenolic content. The chloroform (CHCl_3_) extract showed moderate activity, consistent with its composition of less polar metabolites such as fatty acids and terpenoids. Antibacterial assays revealed extract-specific effects: CHCl_3_ strongly inhibited *Staphylococcus aureus* (18 mm), while EtOAc was more effective against Gram-negative strains, including *Escherichia coli* (18 mm) and *Pseudomonas aeruginosa* (13 mm). Cytotoxicity testing using *Saccharomyces cerevisiae* confirmed that both extracts were non-toxic, maintaining ≥90% cell viability. These findings highlight *P. herculeum* as a novel source of bioactive metabolites with antioxidant and antibacterial potential.

## 1. Introduction

Fungi represent an abundant source of structurally diverse secondary metabolites with remarkable ecological and pharmacological significance [1,2]. Among them, macrofungi have long been recognized as prolific producers of volatile and non-volatile compounds that contribute not only to their survival in complex ecosystems but also to their potential as sources of natural therapeutic agents [3,4]. Mycorrhizal fungi, in particular, play a crucial role in forest ecosystems by forming symbiotic associations with plant roots, which enhance nutrient uptake and strengthen plant tolerance to both biotic and abiotic stresses. These ecological interactions often influence the production of secondary metabolites, many of which exhibit potent biological activities, including antioxidant, antimicrobial, and anticancer properties [5,6,7].

The genus *Phlegmacium* (family Cortinariaceae) includes ectomycorrhizal fungi commonly associated with deciduous and coniferous trees such as *Quercus*, *Pinus*, and *Fagus* species. These fungi contribute significantly to nutrient cycling and soil health, forming part of the ecological balance in forested environments [8,9]. Despite their ecological importance, chemical and biological studies on *Phlegmacium* species remain extremely limited. To date, only a few investigations have explored their metabolomic profiles, and virtually no reports have described the bioactive potential of *Phlegmacium herculeum*, particularly from North African ecosystems. Algeria, with its diverse climatic and ecological zones, offers a unique habitat for a wide variety of macrofungi, yet the chemical diversity and biological potential of its mycorrhizal species remain largely unexplored [10,11,12].

Volatile organic compounds (VOCs) from macrofungi are known to include a broad spectrum of chemical classes such as alcohols, aldehydes, terpenoids, and fatty acids. These compounds are integral to fungal biology, playing essential roles in defense mechanisms by deterring predators and inhibiting competing microorganisms [13,14]. Additionally, VOCs serve as chemical signals facilitating intra- and interspecies communication, thus enabling fungi to adapt and respond dynamically to their environment. Furthermore, these volatiles participate in ecological adaptation processes such as spore dispersal and interactions with plant hosts and other soil organisms, underscoring their multifunctional ecological roles [15,16]. Furthermore, fungi produce several metabolites, including fatty acids, phenolic acids, and organic acids, which are primarily responsible for many of their pharmacological properties. These metabolites exhibit strong antioxidant activities, scavenging free radicals and protecting cells from oxidative damage. They also possess antibacterial properties, limiting the growth of pathogens, and display cytotoxic effects against cancer cells, making them promising candidates for drug development [17,18,19]. The comprehensive characterization of fungal metabolites not only sheds light on fungal chemical ecology but also opens avenues for the discovery of novel natural products with significant biomedical and agricultural applications [20,21].

The present work aimed to conduct a comprehensive analyses and characterization of metabolites produced by the Algerian mycorrhizal macrofungus *Phlegmacium herculeum* using NMR and GC-MS, alongside the evaluation of its biological activities. Specifically, the research aimed to identify and semi-quantify volatile organic compounds using SPME–GC–MS, and to determine metabolites of the fruiting bodies extracts via NMR and GC-MS. In addition, the antioxidant, antibacterial, and cytotoxicity properties of these extracts were assessed in order to establish correlations between chemical composition and biological potential. Ultimately, this study seeks to highlight the ecological and biotechnological importance of *P. herculeum* as a valuable source of natural bioactive compounds within Algerian forest ecosystems.

## 2. Materials and Methods

### 2.1. Sample Collection and Identification

Fruit bodies of the macrofungus *P. herculeum* were collected in January 2018 by Dr. R. Zatout from the Djebel El Ouahch Forest, Constantine, northeastern Algeria [22]. The specimens were found growing under *Pinus* trees, suggesting a possible ectomycorrhizal association with *Cedrus atlantica* roots. Morphological identification was conducted based on macroscopic characteristics using standard mycological keys [23]. For molecular identification, genomic DNA was isolated from the dried specimens following a modified CTAB extraction method. The internal transcribed spacer (ITS) rDNA region was amplified with primers ITS1F and ITS4, and PCR products were sequenced. Resulting sequences were compared to reference sequences in the NCBI GenBank database for taxonomic confirmation and deposited under accession number PQ133121 [24].

### 2.2. Preparation of Extracts of *Phlegmacium herculeum*

The dried fruiting bodies finely powdered (100 g) was extracted twice with chloroform (CHCl_3_) and ethyl acetate (EtOAc). Each extraction involved maceration in 500 mL solvent for 24 h under continuous stirring at ambient temperature. Extracts were filtered and concentrated under reduced pressure at 40 °C using a rotary evaporator. The resulting crude extracts were weighed, stored in sealed glass vials, and kept at 4 °C until further NMR, GC–MS analyses and biological assays [25].

### 2.3. SPME–GC–MS Analysis of Volatile Compounds 

The freshly harvested fruit bodies were cleaned, cut and air-dried at room temperature and finally pulverized. To investigate the volatile chemical profile of *P. herculeum*, a small amount of the powdered sample (about 0.5 g) was placed inside a 4 mL glass vial with PTFE-coated silicone septum. For the extraction of volatiles, a SPME device from Supelco (Bellefonte, PA, USA) with 1 cm fiber coated with 50/30 μm DVB/CAR/PDMS (divinylbenzene/carboxen/polydimethylsiloxane) was used. After reaching the equilibration phase (at 60 °C for 15 min), the fiber was exposed to the headspace of the samples for 20 min to capture and concentrate the volatiles. Lastly, the analytes were desorbed thermally in the GC injector maintained at 250 °C for 2 min in splitless mode. The analysis was carried out on Clarus 500 model Perkin Elmer (Waltham, MA, USA) gas chromatograph coupled with a mass spectrometer equipped. The capillary column was a Varian Factor Four VF-5. To characterize the volatile composition of the sample, the operative conditions were set as follows: from 45 °C to 220 °C at 6°/min and finally held for 10 min. Helium was used as carrier gas at a constant rate of 1 mL/min. MS scans were recorded within the range 35–500 *m*/*z* using EI ionization (energy 70 eV). Identification of compounds was based on the comparison of the mass spectra of pure components stored in the Wiley 2.2 and Nist 11 libraries database and on the comparison of the Linear Retention Indices (LRIs) calculated using a series of alkane standards (C_8_–C_25_ *n*-alkanes) with that available retention data reported in the literature. The relative amounts of the identified components were calculated as percentages of the signal peak area to the total peak area. No internal standards or correction factors were applied. All analyses were performed in triplicate.

### 2.4. NMR and GC–MS Characterization of Crude Extracts

^1^H NMR spectra of the crude extracts were recorded on a Bruker (Karlsruhe, Germany) Anova Advance spectrometer at 400 MHz. Deuterated methanol was used as solvent and as internal standard. All the solvents and reagents were supplied by Sigma-Aldrich Co. (St. Louis, MO, USA).

GC-MS analyses were performed with an Agilent 6850 GC (Milan, Italy) on crude extracts after trimethylsilylation with *N*,*O*-bis(trimethylsilyl)trifluoroacetamide (BSTFA) (Fluka, Buchs, Switzerland) as previously reported [26]. The compounds present in the organic extracts were identified by comparing their EI mass spectra at 70 eV with mass spectra collected in the NIST 20 mass spectral library (available at https://www.nist.gov/srd/nist-standard-reference-database-1a; accessed on 14 October 2025). Moreover, the identification was supported by the LRIs calculated for each analyte as reported in the previous section.

### 2.5. Antioxidant Assay

The antioxidant capacity of *P. herculeum* organic extracts, prepared with chloroform (CHCl_3_) and ethyl acetate (EtOAc), was evaluated in vitro via the DPPH (2,2-diphenyl-1-picrylhydrazyl) free radical scavenging assay. Ascorbic acid (Vitamin C) served as the positive control. A fresh 0.1 mM DPPH solution was prepared by dissolving 0.91 mg of DPPH powder (Sigma-Aldrich) in 23 mL of absolute ethanol (EtOH, analytical grade), ensuring complete dissolution and radical stability. The fungal extracts were emulsified in a 1:1 (*v/v*) mixture of 0.1 M acetate buffer (pH 5.5) and ethanol to enhance dispersion and miscibility.

Starting from an initial concentration of 8 mg/mL, each extract was subjected to five successive two-fold (1:2) serial dilutions, reaching a final test concentration equivalent to 2^−8^ of the original stock, thereby covering a broad dynamic range for evaluating dose-dependent antioxidant effects. The assay was performed in a 96-well microplate format, where 100 µL of the DPPH solution was mixed with 100 µL of each extract dilution or control solution. Each experimental condition, including blanks and controls, was performed in triplicate to ensure reproducibility.

The plate was incubated at room temperature (25 ± 2 °C) for 30 min in the dark to prevent photodegradation of the DPPH radical. Following incubation, the decrease in absorbance was measured at 517 nm using a UV-Visible microplate spectrophotometer. The radical scavenging activity (RSA) was expressed as a percentage of inhibition, calculated using the following equation:

% Inhibition = ((A_0_ − A_s_)/A_0_) × 100

where A_0_ is the absorbance of the DPPH control (without extract), and A_s_ is the absorbance in the presence of the fungal extract.

The IC_50_ values (the concentration required to inhibit 50% of DPPH radicals) were determined graphically by plotting the percentage of inhibition against the logarithm of extract concentration. Vitamin C was used under the same conditions as a positive control to benchmark the antioxidant capacity of each tested plant lipid extract under standardized experimental conditions [27,28].

### 2.6. Antibacterial Assay

The antibacterial activity of the two organic extracts chloroform (CHCl_3_) and ethyl acetate (EtOAc) of *P. herculeum* was evaluated using the standard disk diffusion method with slight modifications [29]. Four bacterial strains were selected for testing: *Escherichia coli* (NCTC 10538), *Staphylococcus aureus* (ATCC 6538), *Pseudomonas aeruginosa* (NCIMB 8626), and *Bacillus spizizenii* (ATCC 6633). Each strain was cultured in Mueller–Hinton broth and incubated at 37 °C for 18–24 h. The bacterial suspensions were then adjusted to match the 0.5 McFarland standard, corresponding to approximately 1.5 × 10^8^ CFU/mL.

Sterile filter paper disks (6 mm diameter) were impregnated with 10 µL of each fungal extract at concentrations of 25 and 50 mg/mL. The extracts were dissolved in DMSO, which was also used alone in negative control disks. All disks were dried under sterile conditions in a laminar flow cabinet. Mueller–Hinton agar plates were inoculated by evenly swabbing the surface with the standardized bacterial suspension using a sterile cotton swab. The extract-impregnated disks, along with a negative control disk (containing only the respective solvent), were placed onto the inoculated plates. All disks were gently pressed to ensure full contact with the agar surface. The plates were then incubated at 37 °C for 24 h.

After incubation, antibacterial activity was assessed by measuring the diameter of the inhibition zones (including the disk diameter) in millimeters (mm). All experiments were conducted in triplicate, and results were expressed as mean ± standard deviation (SD).

### 2.7. Cytotoxicity Assay

The growth-inhibitory potential of the two organic extracts chloroform (CHCl_3_) and ethyl acetate (EtOAc) of *P. herculeum* was evaluated against *Saccharomyces cerevisiae* using a semi-quantitative spot assay. Stock solutions of each extract were prepared in sterile distilled water containing 1% dimethyl sulfoxide (DMSO) to a final concentration of 8 mg/mL, followed by five two-fold serial dilutions to obtain a final concentration range from 2^−2^ to 2^−8^ of the initial stock, thereby covering a broad gradient of exposure.

A yeast cell suspension was prepared from an overnight culture grown in YPD broth. The cells were then washed and resuspended in 100 mM potassium phosphate buffer (pH 7.0) to a final concentration of 2 × 10^7^ cells/mL. For the assay, equal volumes (1:1) of the yeast suspension and each extract dilution were mixed in the wells of a sterile, flat-bottom 96-well microplate. The mixtures were incubated at 37 °C for 30 min to allow interaction between the extract and the yeast cells.

After incubation, 3.0 µL of each treated culture was carefully spotted onto YPD agar plates containing 2% glucose—either supplemented with the corresponding extract concentration or left untreated (for recovery assessment). Control wells containing only yeast cells without fungal extract served as the negative control. The plates were incubated at 37 °C for 48 h, after which yeast growth was documented using a flatbed scanner (Epson^®^ V370, Epson, Suwa, Japan).

The extent of cytotoxicity or growth inhibition was assessed visually by comparing spot intensity and colony formation in treated samples relative to the control. This assay enabled the determination of growth inhibition thresholds and cytotoxic effects of the CHCl_3_ and EtOAc extracts across a defined concentration gradient under standardized conditions [30].

### 2.8. Statistical Analysis

All experiments were performed in triplicate, and data are presented as mean ± standard deviation (SD). Differences between groups were assessed using one-way ANOVA followed by Tukey’s post hoc test, performed with GraphPad Prism 9.0 version 9.0 (GraphPad Software, Boston, MA, USA). A *p*-value less than 0.05 was considered statistically significant

## 3. Results and Discussion

### 3.1. Morphological, Molecular, and Taxonomic Description of the Macrofungus

**Figure 1:**
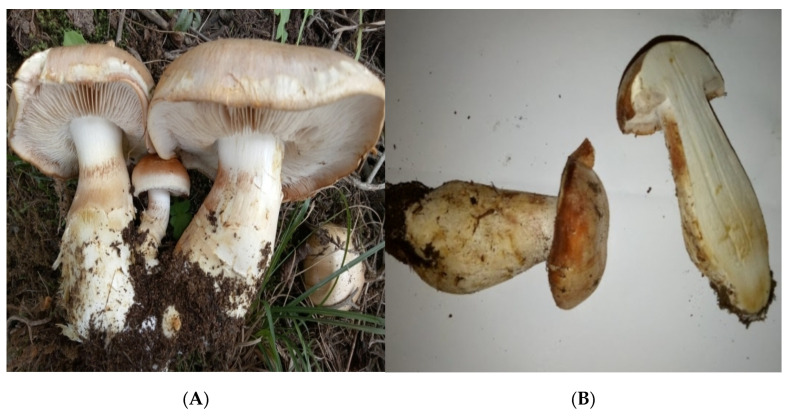
Basidiomata of *Phlegmacium herculeum* at different developmental stages. Photographs by R. Zatout (January 2018) (**A**); Longitudinal section of the basidioma showing the internal structure (cross-section), displayed beside the intact lower part (external view) (**B**).

The collected basidiomata of *P. herculeum* (formerly *Cortinarius herculeus*) (Figure 1) were medium-sized, with convex to plano-convex pilei measuring 3–6 cm in diameter, displaying a cream to brown coloration when fresh, fading to white cream upon drying. The lamellae were adnate to slightly decurrent, initially pale cream and becoming lighter with age. The stipe was cylindrical, 4–8 cm long and 0.5–1 cm thick, with a fibrillose surface and faint cream tones toward the apex. The context was firm, whitish to pale cream, and emitted a faint fungal odor. The pileus surface was viscous when moist [31,32].

To corroborate the morphological observations, molecular characterization was performed by analyzing the internal transcribed spacer (ITS) region of ribosomal DNA. The ITS sequence obtained from the Algerian specimen showed a high similarity (≥99%) with reference sequences of *P. hertulceum* deposited in the NCBI GenBank database under number session PQ133121.

This represents, to our knowledge, the first molecularly confirmed record of *P. herculeum* from Algeria, expanding the known geographical distribution of this species to North Africa. Its occurrence in this region contributes valuable data to the biogeography of *Phelgmacium* species and suggests that North African coniferous forests may harbor greater fungal diversity than previously reported [33].

### 3.2. Analysis of Volatile Compounds by SPME–GC–MS

By SPME-GC-MS twenty-one compounds were detected and identified (Table 1). Propanedioic acid propyl-; (17.5%), 3-methylbutanoic acid (12.6%) and 3-methylbutanoic acid (10.5%) were the most abundant volatiles. 2-Methylbutanal (8.0%), nonadecane (7.8%), 3-methylbutanal (7.2%), *cis*-*β*-ocimene (6.4%) and *trans*-1,10-dimethyl-*trans*-9-decalinol (5.1%) were present with a relevant percentage mean value. The other compounds detected covered a relative amount range from 0.5% to 3.8%.

The constituent propyl propanedioate, followed by 3-methylpentanoic acid and 3-methylbutanoic acid, represent short-chain fatty acids known as fungal metabolites that contribute to the characteristic mushroom-like aroma [34,35] which have been reported to exhibit antimicrobial potential in several basidiomycetes [36].

Among aldehydes, 2-methylbutanal and 3-methylbutanal were the most abundant. These branched aldehydes, which originate from amino acid catabolism, are responsible for pleasant fruity odors and have been detected in other species of *Cortinarius* and *Russula*, suggesting their possible ecological and defensive roles [37,38,39].

Hydrocarbons such as nonadecane and 2,3,5,8-tetramethyldecane were also detected at notable levels. These long-chain alkanes are commonly associated with the outer tissue layer of fruiting bodies and may function as protective agents against desiccation and environmental stress [40].

Terpenoid compounds, including *cis*-*β*-ocimene, limonene, 1,8-cineole, elemol, and farnesane, were also identified. The presence of these mono- and sesquiterpenes is consistent with previous findings in *Phlegmacium* species, reflecting their metabolic capacity to synthesize volatile terpenes with potential ecological and biological significance [41,42].

Additionally, the oxygenated sesquiterpenoid *trans*-1,10-dimethyl-*trans*-9-decalinol and the phenolic compound 3,5-di-*tert*-butylphenol were found. The latter has been described as a potent antioxidant and antimicrobial agent in various fungi, suggesting a possible protective role in *P. herculeum* [43,44].

Overall, the volatile composition of *P. herculeum* reveals a chemically diverse mixture dominated by fatty acids and aldehydes, complemented by significant contributions from terpenes and hydrocarbons. Such diversity indicates a high degree of metabolic plasticity and may reflect adaptive responses to its ecological niche.

Notably, the specimens were collected from Djebel El Ouahch Forest (Constantine, northeastern Algeria), where the fungus grows beneath *Cedrus atlantica*. This mountainous, humid, and organic-rich environment provides favorable conditions for the biosynthesis of a wide range of volatiles. The predominance of fatty acids, aldehydes, and terpenoids may result from metabolic interactions between the fungus, its host plants, and soil microorganisms [45,46].

These findings support the hypothesis that local environmental factors—especially vegetation type and organic matter content—strongly influence the volatile composition of basidiomycetes, endowing them with unique chemical signatures and potential biological activities.

### 3.3. Characterization of the Crude Extracts

The ^1^H NMR spectra of the CHCl_3_ and EtOAc extracts of *P. herculeum* (Figures S1 and S2) revealed signals indicating the presence of several metabolites belonging to different classes of natural compounds. The organic extracts were also analyzed via GC-MS after derivatization with *N*,*O*-bis(trimethylsilyl)trifluoroacetamide (BSTFA) to enhance volatility and improve detection of specific compounds present in the samples. As can be seen from Table 2, the list of compounds detected in the crude extracts includes several organic acids (e.g., lactic acid, succinic acid, fumaric acid) and fatty acids (e.g., palmitic acid, linoleic acid).

### 3.4. Antioxidant Activity

**Figure 2:**
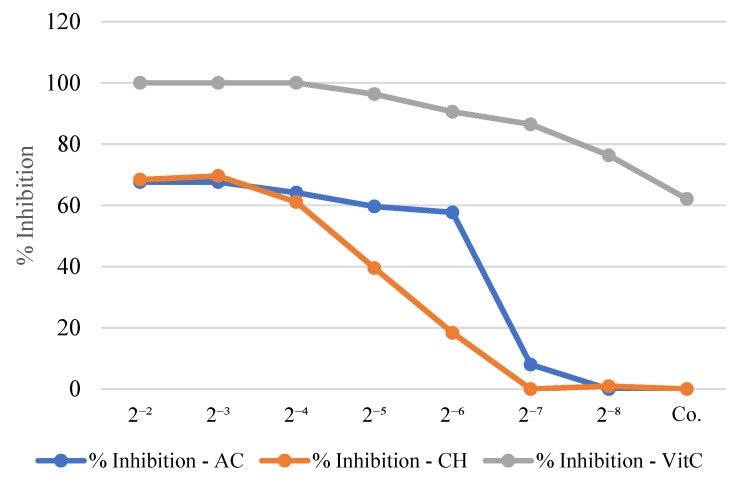
Percentage inhibition of DPPH radical by chloroform (CHCl_3_), ethyl acetate (EtOAc) extracts, and vitamin C across different concentrations.

Both the chloroform (CHCl_3_) and ethyl acetate (EtOAc) extracts exhibited dose-dependent activity, as shown by the progressive increase in radical inhibition with increasing concentrations (Figure 2). Among them, the EtOAc extract demonstrated the strongest antioxidant effect, achieving the highest DPPH scavenging percentage at 8 mg·mL^−1^ and the lowest IC_50_ value, indicating greater hydrogen- or electron-donating capacity [34].

This enhanced activity correlates with its rich composition of phenolic and aromatic compounds, particularly protocatechuic acid and 4-hydroxybenzoic acid, which are known for their free radical-stabilizing properties. Additional polar metabolites, including hydroxy acids and phenylacetic derivatives, may also contribute synergistically to the overall antioxidant effect [47].

In contrast, the CHCl_3_ extract exhibited moderate scavenging potential, consistent with its content of less polar compounds such as long-chain fatty acids (e.g., palmitic and linoleic acids) and dicarboxylic acids, which generally show weaker radical-quenching activity. Its volatile fraction includes terpenoids (α-pinene, camphene, limonene), phenolic volatiles (2,4-di-*tert*-butylphenol), and minor ketones and aldehydes. Although these are less potent than polar phenolics, they may still contribute to antioxidant capacity through rapid radical interactions or transient stabilization [48,49,50].

These results align with previous findings on basidiomycetes, where phenolic metabolites are the primary contributors to antioxidant activity. While the activity was lower than that of ascorbic acid, *P. herculeum*—particularly its EtOAc-soluble fraction—emerges as a promising natural source of antioxidant agents [51,52].

### 3.5. Antibacterial Activity

The antibacterial activity of *P. herculeum* extracts was assessed against four bacterial strains: *Escherichia coli* (NCTC 10538), *Pseudomonas aeruginosa* (NCIMB 8626), *Bacillus spizizenii* (ATCC 6633), and *Staphylococcus aureus* (ATCC 6538) using the agar well diffusion method. The inhibition zones varied according to the extract type and bacterial strain (Table 3).

The chloroform (CHCl_3_) extract showed strong antibacterial effects, particularly against Gram-positive bacteria like *Staphylococcus aureus*, with an inhibition zone of approximately 18 mm. This activity can be attributed to several non-polar and semi-polar compounds identified in the extract, including benzoic acid, phenylacetic acid, palmitic acid, and linoleic acid, all known for their ability to disrupt bacterial membranes and inhibit growth [42]. Fatty acids such as palmitic acid and linoleic acid also contribute significantly due to their well-documented antimicrobial activities by disrupting membrane integrity and function [53]. Additionally, volatile components such as terpenoids and minor phenolic compounds may enhance this antibacterial effect by increasing membrane permeability or inducing stress responses in bacterial cells [54,55].

Conversely, the ethyl acetate (EtOAc) extract exhibited more pronounced activity against Gram-negative bacteria, with inhibition zones of around 18 mm for *E. coli* and 13 mm for *P. aeruginosa*. This effect aligns with the presence of polar phenolic acids like protocatechuic acid, 4-hydroxybenzoic acid, and 4-hydroxyphenylacetic acid, which possess functional groups capable of interfering with bacterial enzymes, cell wall synthesis, and other vital metabolic pathways [56,57]. Although volatile compounds are present in this extract, their contribution to antibacterial activity appears less dominant compared to phenolic metabolites.

Overall, both extracts demonstrate significant antibacterial potential, reflecting a broad spectrum of bioactive metabolites. The combined action of non-polar, polar, and volatile compounds likely contributes to their complementary antibacterial mechanisms, varying with the bacterial strain. These results support *P. herculeum* as a promising source of natural antibacterial agents for further research and development [58,59].

### 3.6. Cytotoxicity Activity

The cytotoxic effects of both the CHCl_3_ and EtOAc extracts were evaluated using a spot-test assay on *Saccharomyces cerevisiae* across dilutions ranging from 2^−2^ to 2^−8^ compared to an untreated control (Table 4). In contrast to preliminary qualitative observations, the quantitative viability assay (Table 4) clearly demonstrates that both extracts maintained 100% cell viability at all tested concentrations, with mortality values ranging between 0–10%, indicating no detectable cytotoxicity under the tested conditions.

These results confirm that the extracts do not exert nonspecific cytotoxic effects on eukaryotic cells such as *S. cerevisiae*. Importantly, the absence of cytotoxicity does not contradict their potential relevance in anticancer research. In fact, several studies emphasize that natural products with low general cytotoxicity are highly valuable because they may act through selective anticancer mechanisms, such as modulation of signaling pathways, inhibition of angiogenesis, antioxidant activity, or enhancement of apoptosis specifically in malignant cells while sparing healthy tissue [60,61].

This selective behavior is widely documented for compounds such as adenosine and fatty acids including linoleic and azelaic acids, which show anticancer selectivity without broad cytotoxicity toward noncancerous cells [62,63,64].

## 4. Conclusions

This research represents a significant step in the molecular identification of *Phlegmacium herculeum* in Algeria, expanding knowledge of its geographical distribution and emphasizing the importance of molecular tools for accurate fungal taxonomy. Chemical analyses revealed a wide diversity of volatile and non-volatile compounds, many of which possess notable biological activities. The strong antioxidant potential demonstrated by the extracts indicates the species’ capacity to mitigate oxidative stress, supporting its possible use in health-related applications. Antibacterial assays confirmed its inhibitory effect against several clinically relevant bacterial strains, underscoring its promise as a natural source for antimicrobial agents. Cytotoxicity evaluation showed that the extracts did not exhibit harmful effects under the tested conditions. This indicates a safe profile for the extracts and supports their suitability for further biological studies, without implying direct anticancer activity.

Overall, *P. herculeum* stands as a valuable source of bioactive metabolites with potential pharmaceutical and biotechnological applications. Further research should focus on isolating and characterizing the active compounds, exploring their molecular mechanisms, and evaluating their safety and efficacy in more complex models. Such comprehensive studies could pave the way for the development of innovative natural compounds that contribute effectively and safely to modern drug discovery.

## Figures and Tables

**Table 1:** List of volatile compounds of *Phelgmacium herculeum* by SPME–GC–MS (percentage mean values).

N.	Component ^1^	LRI_calc_^2^	LRI_lit_^3^	Area %
1	Butanal	568	570	3.8
2	3-Methylbutanal	631	629	7.2
3	2-Methylbutanal	638	639	8.0
4	Pentanal	672	696	0.5
5	3-Methylbutanoic acid	835	839	10.5
6	3,5-Dimethyloctane	922	928	1.4
7	3-Methylpentanoic acid	938	941	12.6
8	Limonene	1021	1029	1.6
9	1,8-Cineole	1035	1033	1.2
10	*cis*-*β*-Ocimene	1040	1037	6.4
11	2-Methyldecane	1062	1059	1.1
12	Dodecane	1210	1214	1.9
14	Propanedioic acid, propyl-	1259	1266	17.5
13	2,3,5,8-Tetramethyldecane	1315	1318	3.3
16	Farnesan	1378	1381	1.9
15	*trans*-1,10-Dimethyl-*trans*-9-decalinol	1418	1423	5.1
17	2(4*H*)-Benzofuranone, 5,6,7,7a-tetrahydro-4,4,7a-trimethyl-	1521	1525	2.6
18	Elemol	1537	1543	1.7
19	Hexadecane	1610	1612	1.6
20	Nonadecane	1907	1910	7.8
21	3,5-Di-*tert*-butylphenol	2314	2310	2.3
	SUM			100.0
	Monoterpenes			8.0
	Monoterpenoids			1.2
	Sesquiterpenes			1.9
	Sesquiterpenoids			6.8
	Aldehydes			19.5
	Fatty acids			23.1
	Hydrocarbons			17.1
	Phenols			2.3
	Others			20.1

^1^ The components are reported according to their elution order on apolar column; ^2^ Linear Retention Indices measured on apolar column; ^3^ Linear Retention indices from the literature.

**Table 2:** List of compounds detected via GC-MS after trimethylsilylation in CHCl_3_ and EtOAc extracts of *P. herculeum.* LRI represents linear retention index and TMS is the trimethylsilyl function, (CH_3_)_3_Si-.

Compound	LRI	Area %	
		CHCl_3_ Extract	EtOAc Extract
Lactic Acid, 2TMS	1066		4.61
β-Lactate 2TMS	1157		0.66
3-Hydroxyisobutyric acid, 2TMS	1173		2.32
Benzoic Acid, TMS	1252	0.87	
Glycerol, 3TMS	1281	2.25	19.37
Phenylacetic acid, TMS	1308	4.25	1.46
Succinic acid, 2TMS	1315	0.55	17.18
Fumaric acid, 2TMS	1353		4.50
Pimelic acid, 2TMS	1604		1.20
4-Hydroxybenzoic acid, 2TMS	1635		3.29
4-Hydroxyphenylacetic acid, 2TMS	1646		1.41
Suberic acid, 2TMS	1695	1.05	7.74
Azelaic acid, 2TMS	1799	17.72	18.82
Protocatechoic acid, 3TMS	1829		1.12
Sebacic acid, 2TMS	1899	1.43	
Palmitic acid, TMS	2042	15.07	
Linoleic acid, TMS	2214	46.85	
Uridine, 3TMS	2487		3.29
9,18-Dihydroxyoctadecanoic acid, 3TMS	2533	2.12	
9,12,13-Trihydroxyoctadec-15-enoic acid, 4TMS	2643	7.83	
Adenosine, 4TMS	2669		13.03

**Table 3:** Antibacterial activity of *Phlegmacium herculeum* extracts (CHCl_3_ and EtOAc) against selected bacterial strains.

Inhibition Zones (mm)		
Bacterial Strain	CHCl_3_ Extract	EtOAc Extract
*Escherichia coli* (NCTC 10538)	10 ± 0.5	18 ± 0.7
*Pseudomonas aeruginosa* (NCIMB 8626)	9 ± 0.4	13 ± 0.6
*Bacillus spizizenii* (ATCC 6633)	-	-
*Staphylococcus aureus* (ATCC 6538)	18 ± 0.8	11 ± 0.5

Values are expressed as mean ± standard deviation (n = 3). “-“ indicates no inhibition zone detected. CHCl_3_: chloroform extract; EtOAc: ethyl acetate extract. Inhibition zones were measured in millimeters (mm).

**Table 4:** Assessment of the effect of CHCl_3_ and EtOAc extracts on cell viability and mortality.

	CHCl_3_		EtOAc	
Concentration (mg/mL)	Viability (%)	Mortality (%)	Viability (%)	Mortality (%)
8	100%	0%	90%	10%
4	100%	0%	100%	0%
2	100%	0%	100%	0%
1	100%	0%	100%	0%
0.5	100%	0%	100%	0%
0.25	100%	0%	100%	0%

## Data Availability

The original contributions presented in this study are included in the article/Supplementary Material. Further inquiries can be directed to the corresponding authors.

## Supplementary Materials

The following supporting information can be downloaded at https://www.mdpi.com/article/10.3390/jof11120894/s1: Figure S1. ^1^H NMR spectrum of chloroform extract, recorded at 400 MHz in CD_3_OD; Figure S2. ^1^H NMR spectrum of ethyl acetate extract, recorded at 400 MHz in CD_3_OD.

## Abbreviations

The following abbreviations are used in this manuscript:

*P.**herculeum*	*Phlegmacium herculeum*
CHCl_3_, CH	Chloroform
EtOAc, AC	Ethyl acetate
VitC	Vitamin C
Co.	Concentration

## References

[1] Bills G.F., Gloer J.B. (2016). Biologically active secondary metabolites from the fungi. Microbiol. Spectr..

[2] Zatout R. (2021). Potential Bio-Control Substances Produced by Fungi and Plants of Different Mediterranean Basin Ecosystems. Doctoral Dissertation.

[3] Salwan R., Katoch S., Sharma V. (2021). Recent developments in shiitake mushrooms and their nutraceutical importance. Fungi in Sustainable Food Production.

[4] Sun X., Shi Y., Shi D., Tu Y., Liu L. (2024). Biological activities of secondary metabolites from the edible-medicinal macrofungi. J. Fungi.

[5] Diagne N., Ngom M., Djighaly P.I., Fall D., Hocher V., Svistoonoff S. (2020). Roles of arbuscular mycorrhizal fungi on plant growth and performance: Importance in biotic and abiotic stressed regulation. Diversity.

[6] Zhao Y., Cartabia A., Lalaymia I., Declerck S. (2022). Arbuscular mycorrhizal fungi and production of secondary metabolites in medicinal plants. Mycorrhiza.

[7] Khan Y., Shah S., Tian H. (2022). The roles of arbuscular mycorrhizal fungi in influencing plant nutrients, photosynthesis, and metabolites of cereal crops—A review. Agronomy.

[8] Liimatainen K., Niskanen T., Dima B., Kytövuori I., Ammirati J.F., Frøslev T.G. (2014). The largest type study of *Agaricales* species to date: Bringing identification and nomenclature of *Phlegmacium* (*Cortinarius*) into the DNA era. Persoonia.

[9] Mrak T., Brailey-Crane P.A., Šibanc N., Martinović T., Gričar J., Kraigher H. (2025). Mycelial communities associated with *Ostrya carpinifolia*, *Quercus pubescens* and *Pinus nigra* in a patchy Sub-Mediterranean Karst woodland. Mycorrhiza.

[10] Mesfek F., Fortas Z., Dib S. (2021). Inventory and ecology of macrofungi and plants in a North-Western Algerian forest. J. Biodivers. Conserv. Bioresour. Manag..

[11] Ait Hamadouche Y., Soulef D.I.B., Mechouet O. (2024). Mycochemical contents of higher fungi and their in vitro effect on plant growth. Not. Bot. Horti Agrobot. Cluj-Napoca.

[12] Zatout R., Cimmino A., Cherfia R., Chaouche N.K. (2021). Isolation of tyrosol the main phytotoxic metabolite produced by the edible fungus *Agaricus litoralis*. Egypt. J. Chem..

[13] Minerdi D., Maggini V., Fani R. (2021). Volatile organic compounds: From figurants to leading actors in fungal symbiosis. FEMS Microbiol. Ecol..

[14] El Jaddaoui I., Rangel D.E., Bennett J.W. (2023). Fungal volatiles have physiological properties. Fungal Biol..

[15] Effmert U., Kalderás J., Warnke R., Piechulla B. (2012). Volatile mediated interactions between bacteria and fungi in the soil. J. Chem. Ecol..

[16] Hung R., Lee S., Bennett J.W. (2015). Fungal volatile organic compounds and their role in ecosystems. Appl. Microbiol. Biotechnol..

[17] Heleno S.A., Martins A., Queiroz M.J.R., Ferreira I.C. (2015). Bioactivity of phenolic acids: Metabolites versus parent compounds: A review. Food Chem..

[18] He M., Peng Q., Xu X., Shi B., Qiao Y. (2024). Antioxidant capacities and non-volatile metabolites changes after solid-state fermentation of soybean using oyster mushroom (*Pleurotus ostreatus*) mycelium. Front. Nutr..

[19] Sureshkumar S., Merlin I., Prasai J.R., Rajapriya P., Pandi M. (2022). Antioxidant, antibacterial, cytotoxicity, and phytochemical potentials of fungal bioactive secondary metabolites. J. Basic Microbiol..

[20] Wadhwa K., Kapoor N., Kaur H., Abu-Seer E.A., Tariq M., Siddiqui S., Alghamdi S. (2024). A comprehensive review of the diversity of fungal secondary metabolites and their emerging applications in healthcare and environment. Mycobiology.

[21] Bhattarai K., Kabir M.E., Bastola R., Baral B. (2021). Fungal natural products galaxy: Biochemistry and molecular genetics toward blockbuster drugs discovery. Adv. Genet..

[22] Zatout R., Christopher B., Cherfia R., Chaoua S., Chaouche N.K. (2023). A new record of *Agaricus litoralis*, a rare edible macro-fungus from Eastern Algeria. Mycopath.

[23] Bon M. (1987). Guía de Campo de los Hongos de Europa.

[24] Zatout R., Benslama O., Makhlouf F.Z., Cimmino A., Salvatore M.M., Andolfi A., Masi M. (2025). Exploring the potential of *Genista ulicina* phytochemicals as natural biocontrol agents: A comparative in vitro and in silico analysis. Toxins.

[25] Zatout R., Kacem Chaouche N. (2023). Antibacterial activity screening of an edible mushroom *Agaricus litoralis*. Int. J. Bot. Stud..

[26] Salvatore M.M., Pappalardo C., Suarez E.G.P., Salvatore F., Andolfi A., Gesuele R., Galdiero E., Libralato G., Guida M., Siciliano A. (2024). Ecotoxicological and metabolomic investigation of chronic exposure of *Daphnia magna* (Straus, 1820) to yttrium environmental concentrations. Aquat. Toxicol..

[27] Blois M.S. (1958). Antioxidant determinations by the use of a stable free radical. Nature.

[28] Brand-Williams W., Cuvelier M.E., Berset C.L.W.T. (1995). Use of a free radical method to evaluate antioxidant activity. LWT-Food Sci. Technol..

[29] Balouiri M., Sadiki M., Ibnsouda S.K. (2016). Methods for in vitro evaluating antimicrobial activity: A review. J. Pharm. Anal..

[30] Ben Amor A., Hemmami H., Gherbi M.T., Seghir B.B., Zeghoud S., Gharbi A.H., Barhoum A. (2025). Synthesis of spherical carbon nanoparticles from orange peel and their surface modification with chitosan: Evaluation of optical properties, biocompatibility, antioxidant and anti-hemolytic activity. Biomass Convers. Biorefin..

[31] Liimatainen K., Kim J.T., Pokorny L., Kirk P.M., Dentinger B., Niskanen T. (2022). Taming the beast: A revised classification of Cortinariaceae based on genomic data. Fungal Divers..

[32] Høiland K., Danielsen J.S., Gulden G., Marthinsen G., Tjessem I.V. (2025). Web caps *Cortinarius* and *Phlegmacium* (Agaricales, Cortinariaceae) in Svalbard and on Bear Island. AGARICA.

[33] Blanco O., Crespo A., Divakar P.K., Elix J.A., Lumbsch H.T. (2005). Molecular phylogeny of parmotremoid lichens (Ascomycota, Parmeliaceae). Mycologia.

[34] Gómez I., Lavega González R., Tejedor-Calvo E., Pérez Clavijo M., Carrasco J. (2022). Odor profile of four cultivated and freeze-dried edible mushrooms by using sensory panel, electronic nose and GC-MS. J. Fungi.

[35] Deng G., Li J., Liu H., Wang Y. (2024). Volatile compounds and aroma characteristics of mushrooms: A review. Crit. Rev. Food Sci. Nutr..

[36] Guimarães A., Venâncio A. (2022). The potential of fatty acids and their derivatives as antifungal agents: A review. Toxins.

[37] Tian R., Liang Z.Q., Wang Y., Zeng N.K. (2022). Analysis of aromatic components of two edible mushrooms, *Phlebopus portentosus* and *Cantharellus yunnanensis* using HS-SPME/GC-MS. Results Chem..

[38] Kitajima S., Maruyama Y., Kuroda M. (2023). Volatile short-chain aliphatic aldehydes act as taste modulators through the orally expressed calcium-sensing receptor CaSR. Molecules.

[39] Aisala H., Manninen H., Laaksonen T., Linderborg K.M., Myoda T., Hopia A., Sandell M. (2020). Linking volatile and non-volatile compounds to sensory profiles and consumer liking of wild edible Nordic mushrooms. Food Chem..

[40] Ladygina N., Dedyukhina E.G., Vainshtein M.B. (2006). A review on microbial synthesis of hydrocarbons. Process Biochem..

[41] Farré-Armengol G., Filella I., Llusià J., Peñuelas J. (2017). β-Ocimene, a key floral and foliar volatile involved in multiple interactions between plants and other organisms. Molecules.

[42] Ji W., Ji X. (2021). Comparative analysis of volatile terpenes and terpenoids in the leaves of *Pinus* species—A potentially abundant renewable resource. Molecules.

[43] Rathna J., Bakkiyaraj D., Pandian S.K. (2016). Anti-biofilm mechanisms of 3,5-di-tert-butylphenol against clinically relevant fungal pathogens. Biofouling.

[44] Zhao F., Wang P., Lucardi R.D., Su Z., Li S. (2020). Natural sources and bioactivities of 2,4-di-tert-butylphenol and its analogs. Toxins.

[45] Duc N.H., Vo H.T., van Doan C., Hamow K.A., Le K.H., Posta K. (2022). Volatile organic compounds shape belowground plant–fungi interactions. Front. Plant Sci..

[46] Rocco M., Kammer J., Santonja M., Temime-Roussel B., Saignol C., Lecareux C., Ormeño E. (2025). Is litter biomass a driver of soil volatile organic compound fluxes in Mediterranean forest?. Biogeosciences.

[47] Kim W., Lee B., Park J., Kim H.J., Cheong H. (2018). Comparative antioxidant activity and structural feature of protocatechuic acid and phenolic acid derivatives by DPPH and intracellular ROS. Lett. Drug Des. Discov..

[48] Gülcin I. (2012). Antioxidant activity of food constituents: An overview. Arch. Toxicol..

[49] Szczepańska P., Rychlicka M., Groborz S., Kruszyńska A., Ledesma-Amaro R., Rapak A., Lazar Z. (2023). Studies on the anticancer and antioxidant activities of resveratrol and long-chain fatty acid esters. Int. J. Mol. Sci..

[50] Heer A., Guleria S., Razdan V.K. (2017). Chemical composition, antioxidant and antimicrobial activities and characterization of bioactive compounds from essential oil of *Cinnamomum tamala* grown in north-western Himalaya. J. Plant Biochem. Biotechnol..

[51] Ghasemzadeh A., Jaafar H.Z., Rahmat A. (2010). Antioxidant activities, total phenolics and flavonoids content in two varieties of Malaysia young ginger (*Zingiber officinale* Roscoe). Molecules.

[52] Zatout R., Benarbia R., Yalla I. (2024). Phytochemical profiles and antimicrobial activities of *Phellinus* mushroom: Implications for agricultural health and crop protection. Agric. Res. J..

[53] Ferreira I.C., Barros L., Abreu R. (2009). Antioxidants in wild mushrooms. Curr. Med. Chem..

[54] Burt S. (2004). Essential oils: Their antibacterial properties and potential applications in foods—A review. Int. J. Food Microbiol..

[55] Stephane F.F.Y. (2020). Terpenoids as Important Bioactive Constituents of Essential Oils. Essential Oils: Bioactive Compounds, New Perspectives and Applications.

[56] Cho J.Y., Moon J.H., Seong K.Y., Park K.H. (1998). Antimicrobial activity of 4-hydroxybenzoic acid and *trans* 4-hydroxycinnamic acid isolated and identified from rice hull. Biosci. Biotechnol. Biochem..

[57] Sánchez–Maldonado A.F., Schieber A., Gänzle M.G. (2011). Structure–function relationships of the antibacterial activity of phenolic acids and their metabolism by lactic acid bacteria. J. Appl. Microbiol..

[58] Borges A., Ferreira C., Saavedra M.J., Simões M. (2013). Antibacterial activity and mode of action of ferulic and gallic acids against pathogenic bacteria. Microb. Drug Resist..

[59] Alves M.J., Ferreira I.C., Dias J., Teixeira V., Martins A., Pintado M. (2012). A review on antimicrobial activity of mushroom (Basidiomycetes) extracts and isolated compounds. Planta Medica.

[60] Jia S., Li L., Yu C., Peng F. (2024). Natural products’ antiangiogenic roles in gynecological cancer. Front. Pharmacol..

[61] Oraibi A.I., Dawood A.H., Trabelsi G., Mahamat O.B., Chekir-Ghedira L., Kilani-Jaziri S. (2025). Antioxidant activity and selective cytotoxicity in HCT-116 and WI-38 cell lines of LC-MS/MS profiled extract from *Capparis spinosa* L.. Front. Chem..

[62] Virtanen S.S., Kukkonen-Macchi A., Vainio M., Elima K., Härkönen P.L., Jalkanen S., Yegutkin G.G. (2014). Adenosine inhibits tumor cell invasion via receptor-independent mechanisms. Mol. Cancer Res..

[63] Qiu J., Zhao Z., Suo H., Paraghamian S.E., Hawkins G.M., Sun W., Bae-Jump V. (2024). Linoleic acid exhibits anti-proliferative and anti-invasive activities in endometrial cancer cells and a transgenic model of endometrial cancer. Cancer Biol. Ther..

[64] Dongdong Z., Jin Y., Yang T., Yang Q., Wu B., Chen Y., Zhou F. (2019). Antiproliferative and immunoregulatory effects of azelaic acid against acute myeloid leukemia via the activation of notch signaling pathway. Front. Pharmacol..

