# Supplementary material for: Extracts of the Algerian Fungus Phlegmacium herculeum: Chemical Analysis, Antioxidant, Antibacterial, and Cytotoxicity Evaluation
Source: J Fungi (Basel). 2025 Dec 18;11(12):894. doi: 10.3390/jof11120894 (PMC12733421; doi:10.3390/jof11120894)
Supplement: Supplementary file 1 [file jof-11-00894-s001.zip › jof-4014595-supplementary.pdf]

Supplementary Materials

Extracts of the Algerian Fungus *Phlegmacium herculeum*: Chemical Analysis, Antioxidant, Antibacterial, and Cytotoxicity Evaluation

Roukia Zatout [1,*], Stefania Garzoli [2], Lounis Youcef Khodja [3,4], Ouided Abdelaziz [5], Maria Michela Salvatore [7], Anna Andolfi [6], Marco Masi [6,*] and Alessio Cimmino [6]

[1] Department of Microbial Biotechnology, Faculty of Natural and Life Science, University of Blida 1, Ouled Yaich, Blida-09000, Algeria
[2] Department of Chemistry and Technologies of Drug, Sapienza University, 00185 Rome, Italy. stefania.garzoli@uniroma1.it (S.G.)
[3] Laboratoire de recherche en écologie et en environnement, Faculté des sciences de la nature et de la vie, Université de Béjaia-06000, Béjaia, Algérie; lounisyoucefkhodja@yahoo.fr
[4] Department of Plant Biology and Ecology, University of Sétif 1, Algéria
[5] Laboratoire de biochimie appliqué, Département de biochimie et biologie cellulaire et moléculaire, Faculté des sciences de la Nature et de la Vie, Université frères Mentouri, Constantine 1, Route d'Ain el bey, Constantine, 25017, Algeria; Algeria; az_wided@yahoo.fr (O.A.)
[6] Department of Chemical Sciences, University of Naples Federico II, 80126 Naples, Italy; andolfi@unina.it (A.A.); marco.masi@unina.it (M.M.)
[7] Department of Veterinary Medicine and Animal Production, University of Naples Federico II, 80137 Naples, Italy; mariamichela.salvatore@unina.it
* Correspondence: marco.masi@unina.it (M.M.); roukia.zatout@umc.edu.dz (R.Z.)

Contents

Figure S1. ^{1}H NMR spectrum of chloroform extract, recorded at 400 MHz in CD_3OD.

Figure S2. ^{1}H NMR spectrum of ethyl acetate extract, recorded at 400 MHz in CD_3OD.

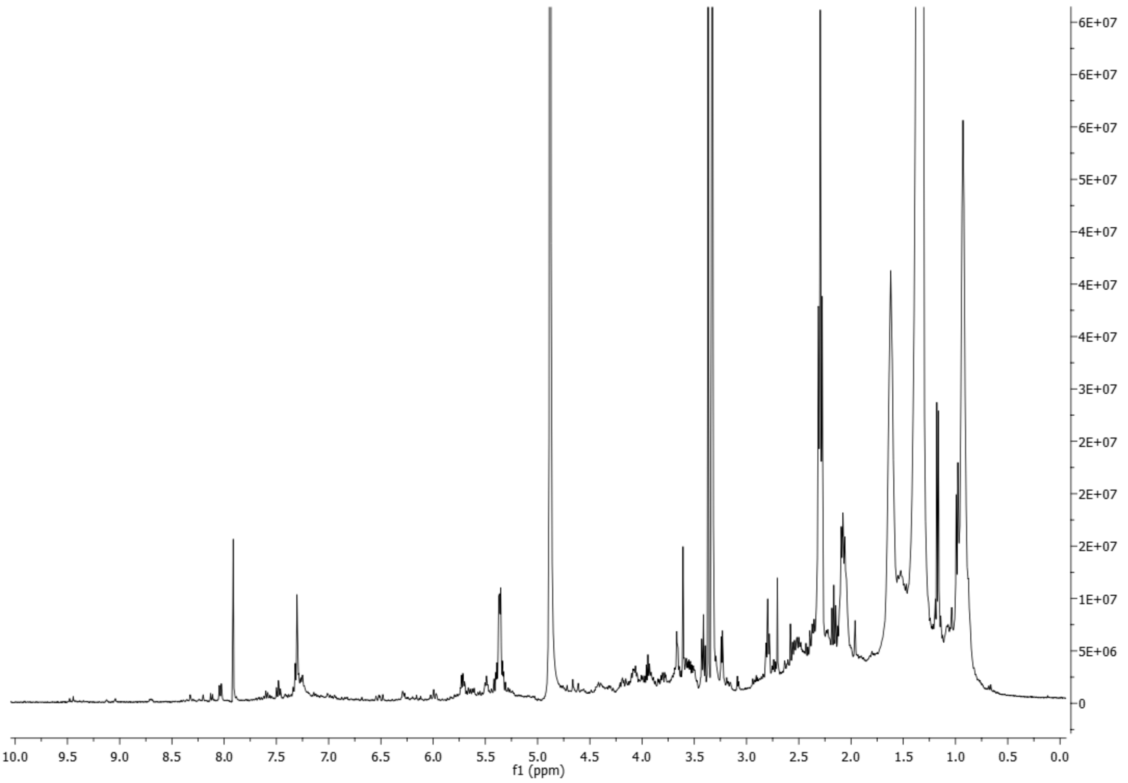

Figure S1. ^{1}H NMR spectrum of chloroform extract, recorded at 400 MHz in CD_3OD.

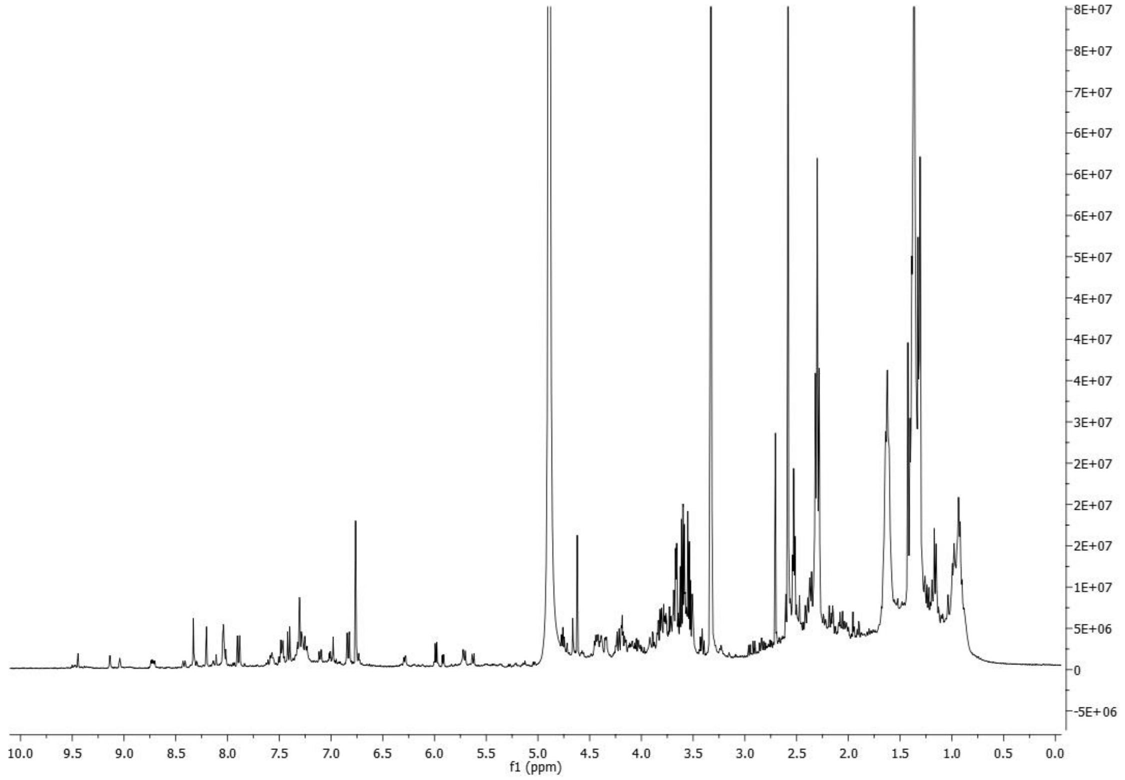

Figure S2. ^{1}H NMR spectrum of ethyl acetate extract, recorded at 400 MHz in CD_3OD.
